# Out-Of-Pocket Expenditures on Dental Care for Schoolchildren Aged 6 to 12 Years: A Cross-Sectional Estimate in a Less-Developed Country Setting

**DOI:** 10.3390/ijerph16111997

**Published:** 2019-06-05

**Authors:** Carlo Eduardo Medina-Solís, Leticia Ávila-Burgos, María de Lourdes Márquez-Corona, June Janette Medina-Solís, Salvador Eduardo Lucas-Rincón, Socorro Aida Borges-Yañez, Miguel Ángel Fernández-Barrera, América Patricia Pontigo-Loyola, Gerardo Maupomé

**Affiliations:** 1 The Academic Area of Dentistry in the Health Sciences Institute, the Autonomous University of the State of Hidalgo, Pachuca 42039, Mexico; lulumaco_1@yahoo.com.mx (M.d.L.M.-C.); mafba.mafb@gmail.com (M.Á.F.-B.); americap@uaeh.edu.mx (A.P.P.-L.); 2 The Center for Advanced Studies and Research in Dentistry “Keisaburo Miyata”, Faculty of Dentistry, the Autonomous University of the State of Mexico, Toluca 50000, Mexico; chavalalo19@hotmail.com; 3 The Center for Health Systems Research, the National Institute of Public Health, Cuernavaca 62100, Mexico; 4 Ministry of Education of Campeche, Sub-secretary of Educational Coordination, Direction of Coordination and Budgetary Management, Campeche 24095, Mexico; medina_sjj@hotmail.com; 5 School of Dentistry, the Ixtlahuaca University Centre, Ixtlahuaca 50080, Mexico; 6 DEPeI School of Dentistry, the National Autonomous University of Mexico, Mexico City 04360, Mexico; aborges@unam.mx; 7 Richard M. Fairbanks School of Public Health, Indiana University/Purdue University in Indianapolis, Indianapolis, IN 46202, USA; gmaupome@iu.edu; 8 The Indiana University Network Science Institute, Bloomington, IN 47408, USA

**Keywords:** oral health, dental care, health expenditure, out-of-pocket expenditure, Mexico

## Abstract

*Aim*: The objective of this study was to estimate the Out-Of-Pocket Expenditures (OOPEs) incurred by households on dental care, as well as to analyze the sociodemographic, economic, and oral health factors associated with such expenditures. *Method*: A cross-sectional study was conducted among 763 schoolchildren in Mexico. A questionnaire was distributed to parents to determine the variables related to OOPEs on dental care. The amounts were updated in 2017 in Mexican pesos and later converted to 2017 international dollars (purchasing power parities–PPP US $). Multivariate models were created: a linear regression model (which modeled the amount of OOPEs), and a logistic regression model (which modeled the likelihood of incurring OOPEs). *Results*: The OOPEs on dental care for the 763 schoolchildren were PPP US $53,578, averaging a PPP of US $70.2 ± 123.7 per child. Disbursements for treatment were the principal item within the OOPEs. The factors associated with OOPEs were the child’s age, number of dental visits, previous dental pain, main reason for dental visit, educational level of mother, type of health insurance, household car ownership, and socioeconomic position. *Conclusions*: The average cost of dental care was PPP US $70.2 ± 123.7. Our study shows that households with higher school-aged children exhibiting the highest report of dental morbidity—as well as those without insurance—face the highest OOPEs. An array of variables were associated with higher expenditures. In general, higher-income households spent more on dental care. However, the present study did not estimate unmet needs across the socioeconomic gradient, and thus, future research is needed to fully ascertain disease burden.

## 1. Introduction

Despite being preventable through cost-effective actions such as tooth brushing and pit and fissure sealants (together with interproximal cleaning techniques such as flossing, a healthy diet and reduced sugar intake), oral diseases such as dental caries and periodontal diseases continue to be the leading oral health problems in Mexico, affecting a large proportion of the population [1,2,3,4]. Several studies have reported high prevalence and incidence of these problems among the general population, implying significant unmet treatment needs [1,2,3,4]. The dmft index (decayed, missing or filled primary teeth in the primary dentition) ranges from 1.00 to 2.20 and exhibits a prevalence of approximately 60% [5]. The DMFT index (decayed, missing or filled permanent teeth refers to dental caries in the permanent dentition) ranges from 0.74 and 1.38 and exhibits a prevalence fluctuating between 36.2% and 52.7% [5]. Such diseases are concentrated in population groups suffering from the worst socioeconomic conditions, including those with the highest burden of dental mortality [2,6].

Dental morbidity is rarely fatal, and is generally not disabling. Because this condition results in few “role exemptions,” there is little incentive to adopt the “sick role” for oral health issues. In addition, given competing priorities in allocating health care resources, people accord little importance to their oral health [7,8,9,10,11,12,13,14]. Coverage for dental services by the Mexican public health system is underdeveloped and mostly limited to caries prevention, placement of pit and fissure sealants, placement of restorations and tooth extractions, leaving out most specialized treatments [15]. This lack of coverage for a substantial proportion of dental care services is aggravated by long wait times in public institutions [16] and might lead to postponing using these services. Among individuals reporting oral health problems, the level of coverage for publicly funded dental services during the year prior to the survey was approximately 50% [8]. Overall, the percentage of Mexican children utilizing dental services varied between 15% and 65% [9,10,11,12,13], with as many as 33.2% of children between six and seven years old having never received any dental services [14].

Various studies have documented that seeking and utilizing dental care services is associated not only with the presence and severity of disease, but also with individual characteristics, the most important of which are sex, age, level of schooling, access to health insurance, and income level or household wealth [8,9,10,11,12,13,14]. The ability of households to pay for these services is an important factor, with those lacking access to publicly funded dental services being at a disadvantage. This acquires particular relevance given that the structure of the Mexican health system is fragmented and driven by employment status [17]. Until 2000, people working in the formal sector (~50 million, or roughly half the population) were entitled to medical and pharmaceutical coverage under the Social Security system, financed by contributions from employees and employers; the rest of the population had limited access. Such large groups comprised the self-employed, the unemployed, agricultural workers, and generally those with low income. This situation led to a problematic situation with regard to OOPEs. OOPE health payments are the money paid by households to purchase private care and medications. In 2003, 58% of total health spending came from household out-of-pocket expenditures [18].

This method of financing health care, which required households to incur out-of-pocket expenditures (OOPEs), led the Mexican government to implement a financial reform of the Mexican health system in 2003 [19]. Its objective was ensuring universal health coverage and providing financial protection to households without Social Security coverage. The creation of the *Seguro Popular* (SP), a voluntary public insurance scheme that offers a package of 294 health interventions to its members, offered a partial solution. The SP is financed primarily by the federal and state governments and, to a lesser extent, by household contributions; it is free of charge for those households in the lowest three income deciles. By 2017, 60.6 million people, or 43.3% of the entire Mexican population, had allegedly become affiliated with the SP [19,20]. This reform aimed to reduce OOPEs and the risk for the poorest population groups of incurring catastrophic health expenses. The SP has had a significant impact on improving access to and utilization of health services, reducing the likelihood of households incurring OOPEs [21]. Notwithstanding these advances, publicly-funded coverage for dental services has not significantly improved; access to most of such services continues to be dependent on the ability of households to pay out-of-pocket. In one of the few studies undertaken in Mexico to analyze OOPEs associated with the use of dental services, Perez et al. [8] found that more than US $684 million (purchasing power parity [PPP]) was spent in Mexico in 2000 in this category, with 8.5% of all households having had at least some expenditure. More than PPP US $406.5 million was spent in 2002, representing 4% of households, and over PPP US $455 million was spent in 2004, amounting to 5% of households. Average expenditures per household were PPP US $85, $104 and $87 dollars in 2000, 2002, and 2004, respectively. Despite the importance of assessing the amount of OOPEs devoted to dental services to fully cover costs, no recent studies have examined this issue.

The objective of this study was to estimate the OOPEs incurred by households on dental care, as well as to analyze the sociodemographic, economic, and oral health factors associated with such expenditures. Our investigation was limited to schoolchildren ages 6 to 12 years who had used dental care services at least once in the 12 months prior to the study.

## 2. Materials and Methods

### 2.1. Design and Study Sample

This cross-sectional study was carried out in 14 public elementary schools in Pachuca City, Mexico. The methodology has been partially reported elsewhere [14,22]. Hidalgo is one of the Mexican states that participated in the fluoridated domestic salt program implemented nationally in 1991. Mexico has no water fluoridation program at the national level; however, some states, including parts of Hidalgo, have water with naturally high levels of fluoride. This is not the case in Pachuca, the capital city of Hidalgo [23]. According to an analysis of data from the 2012 National Survey of Health and Nutrition (ENSANUT 2012), between 57.8% and 84.6% of the Mexican population have an inappropriate level of consumption of added sugars (above the recommended upper limit of >10% of total energy consumed). The proportion of individuals consuming inappropriate levels of added sugar increases to 61.9% to 89.2% in urban areas, and falls to 46.6% to 68.7% in rural areas [24]. 

**Figure 1:**
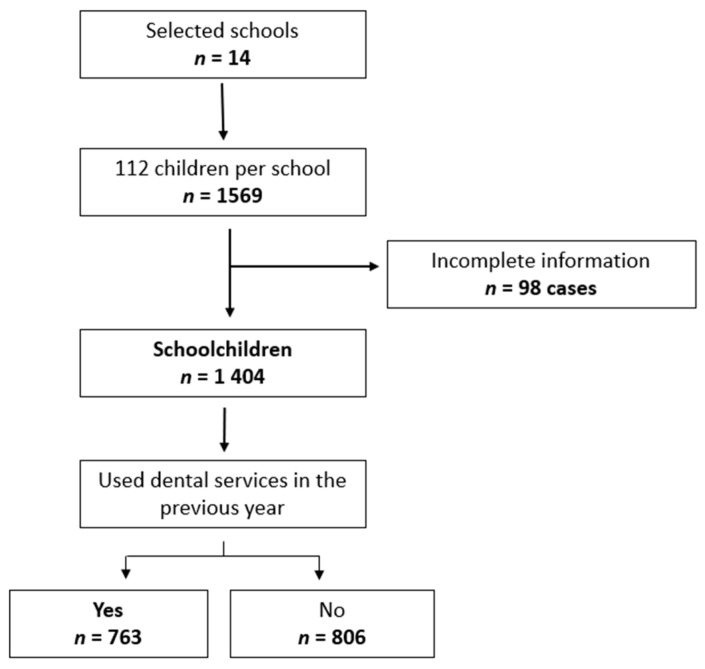
Selection of the sample of schoolchildren from participating schools, Pachuca, Mexico.

The inclusion criteria were (a) either sex; (b) age 6 to 12 years; (c) enrolled in any public elementary school in the study; and (d) parents/guardians had authorized participation in the study and signed the informed consent. Sample size was calculated based on a smallest estimated proportion of 35%; a 95% confidence level; 3% accuracy; and a 10% non-response rate. The estimated sample population was 1554 schoolchildren. In the first stage, a random selection was made of 14 of the 94 public primary schools in the city. In the second stage, a random sample was taken from the enrollment lists to choose potential study participants. A total of 112 children per school were randomly selected, distributed similarly by age and sex, as recommended by the WHO in its sampling strategies [25]. Only 98 cases were eliminated because of missing data, with a resulting total of 1404 subjects enrolled in the study (89.5% of the target population). For the purposes of the present analysis, only those subjects reporting at least one dental visit in the year prior to the survey (*n* = 763 schoolchildren) were included. Figure 1 contains the details of our sample selection.

### 2.2. Data Collection, Variables and Conformation Variables

Sociodemographic, economic, and dental variables were collected by means of a structured questionnaire previously validated in a pilot study. The questionnaire and informed consent forms were completed by the parents/guardians of the sampled children and collected at the schools.

To estimate dental OOPEs during the year prior to the study (our dependent variable), parents/guardians were asked about expenses paid for transportation, consultations, treatments, medications, laboratory tests, radiographic studies, and others. The amounts were reported in updated 2017 Mexican pesos using the consumer price index published by the National Institute of Statistics (INEGI) [26]. This was subsequently converted to 2017 international dollars using a PPP rate of one international dollar equivalent to 9.23 Mexican pesos [27]. The international dollar (PPP US$) is a hypothetical unit calculated by the World Bank that allows adjustment for PPP [28]; it represents the amount of local currency units needed to acquire within the country in question the same amount of goods that would be purchased with one US dollar in the US.

Independent variables were age, sex, number of household members, number of dental visits in the previous year, experiencing dental pain in the previous year, reason for the last dental visit, self-report of oral disease, self-assessment of perceived oral health, tooth brushing frequency, oral health knowledge, maximum schooling levels for both the mother and father, type of health insurance coverage, socioeconomic position (derived from the physical features of the family home including building materials and ownership of household assets such as a refrigerator, stove, microwave, and so on) and whether the household owned a car. We also included a group of 12 variables to explore the different dimensions of knowledge on the part of parents/guardians regarding oral health (the importance of tooth brushing in preventing oral disease, dental checkups, application of fluoride, etc.). One variable related to oral health knowledge on the part of parents/guardians was extracted using principal components analysis (alpha de Cronbach > 0.70); this variable was utilized in a previous study [22]. In addition, two variables indicating socioeconomic position were generated from surveys. Curative services included extraction of teeth or molars, pain relief, fillings (amalgam and resin), dental fracture management and specialized treatment (orthodontics, endodontics, periodontics, prostheses, and surgery). Preventive services included examination, application of fluoride and sealants, and cleaning (prophylaxis).

### 2.3. Statistical Analysis

A univariate analysis was carried out where measures of central tendency and dispersion were calculated for the quantitative variables. Categorical variables were assessed through frequencies for each category as well as by percentage. Bivariate analyses included Mann-Whitney, Kruskal-Wallis, and non-parametric testing for trends (NPTT) to contrast OOPEs across variables. 

We used two multivariate models: a linear regression model (which modeled the amount of OOPEs) expressed as a coefficient with 95% confidence interval (95% CI), and a logistic regression model (which modeled the likelihood of incurring OOPEs) expressed as an odds ratio (OR), also with a 95% confidence interval (95% CI). We also reported the p values, considered statistically significant if they were less than 0.05. To control for confounders, the final model included those variables with a *p* value < 0.25 in the bivariate analysis. We performed a variance inflation factor (VIF) analysis to detect and prevent multicollinearity among independent variables [29,30]. Confidence intervals were calculated with robust standard errors in both models. Since data were collected from children attending elementary schools who shared common characteristics (cluster), we assumed that observations within these clusters could be correlated while observations among clusters could not [31]. Data were analyzed using Stata software version 11.0 (StataCorp LLC, College Station, TX, USA).

### 2.4. Ethical Considerations

Research was conducted in accordance with ethical standards set by the institutional research committee; ethical provisions complied with the Helsinki Declaration and its subsequent amendments or comparable ethical standards. The protocol was approved by the Institutional Review Board of the Autonomous University of the State of Hidalgo (Mexico) (UAEH-DI-ICSA-ODO-CF-016). Written consent was obtained from the parents/guardians of the participating children.

### 2.5. Data Availability Statement

Data is available on request from the authors.

### 2.6. The Mexican Dental Health Care System

The Mexican dental health care system is a mixed and fragmented system comprised of public service and Social Security institutions, third-party payment systems and private carriers. The overwhelming majority of services are delivered under a fee-for-item, out-of-pocket arrangement run by largely unregulated dental professionals and thus at the mercy of the dental market. The public health sector is responsible for a small, essentially unidentified and fluid set of services largely restricted to urban settings. In contrast, dozens of dental school clinics at numerous public and private universities offer dental services to the population at much reduced prices [8], provided by students under close faculty supervision [8]. While not an organized, distinct clinical care system, services delivered in dental school clinics constitute a significant portion of dental care services available to the open population [32].

## 3. Results

Of the 1404 children originally included in the study, we selected for our sample those 763 (54.3%) who had visited the dentist at least once in the year prior to data collection. Table 1 presents demographic and economic characteristics and data related to oral health. The mean age was 8.90 ± 1.96; 51% of the subjects were girls. The average number of household members was 4.40 ± 1.22, and the mean number of dental visits was 2.58 ± 2.36. Of the parents/guardians surveyed, 68.8% reported that their children had experienced dental pain in the previous 12 months. Likewise, most (65.6%) of the children had last been to the dentist for curative care. About a quarter (22.5%) reported having tooth/gum disease, while 48.4% were classified as having good/very good oral health status. The rate of tooth brushing at least once a day was 82.3%. We divided the generated variable concerning oral health knowledge into tertiles; the socioeconomic variables are presented in Table 2. In terms of schooling, 39.2% of mothers had attended high school while 33.5% of parents had attended high school or higher. About a third (30.1%) reported having no health insurance. The variables on socioeconomic position (including housing and household assets) were also divided into tertiles, with 63.2% of households owning a car.

**Figure 2:**
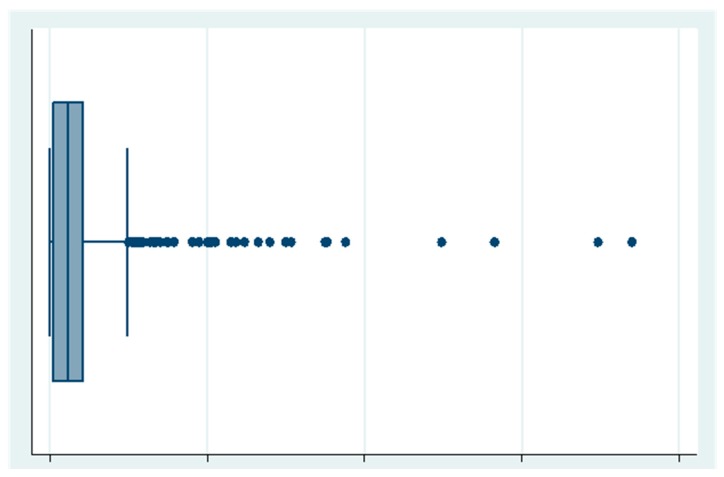
Distribution of OOPEs (Mean: PPP US $70.2 ± 123.7; limits: PPP US $0.00 to PPP US $801.73). The large standard deviation suggests that a considerable proportion of households had no dental OOPEs.

OOPEs for dental care (Figure 2) and its components are shown in Table 3. Household OOPEs for 763 children totaled PPP US $53,578, with an average of PPP US $70.2 ± 123.7 per person. Payment for treatment was the principal expense item (45% of the total; PPP US $31.9 ± 87.4), followed by consultations and medicines.

Table 4 shows that the households with the highest OOPEs were those with the highest number of visits in the previous year (*p* = 0.0002): these expenditures increased significantly (NPTT *p* < 0.001) with the number of visits. The OOPE average for one visit was PPP US $47.3, PPP US $60.0 for two visits, and PPP US $112.5 for three or more visits. OOPEs were significantly higher among those who had experienced oral pain during the 12 months prior to the study versus those who had not (*p* < 0.0001); those who had a poor perception of their health spent more (*p* < 0.001). This expense trend was also significantly higher for those who had received specialized clinical treatment during their last visit to the dentist compared to those who had sought care for curative or preventive reasons (*p* < 0.001).

OOPEs were higher for those with greater oral health knowledge than for those with less adequate knowledge (*p* < 0.01). Households with children with mixed dentition (8/9 years) did not have higher OOPEs than those with children with only primary dentition (6/7 years) or permanent dentition (10/12 years) (*p* = 0.0794). Having more household members was not associated with higher OOPEs (*p* = 0.0792). No differences emerged by sex, self-reporting of disease or brushing frequency (Table 4).

Table 5 shows that parents whose schooling only reached elementary school level had the highest OOPEs (*p* < 0.01), whereas children whose mothers had attended middle school were reported as having the highest OOPEs (*p* = 0.061). Households lacking health insurance had the highest (PPP US $85.2 ± 149.4) followed by those with Social Security coverage (PPP US $74.3 ± 127.9). The lowest OOPEs were observed among SP affiliates (PPP US $32.0 ± 43.3) (*p* < 0.01). Households owning a car had higher OOPEs than those without a car (*p* < 0.001). The other two variables indicating socioeconomic position (housing and household assets) were also statistically significant (*p* < 0.001) and showed a positive trend (*p* < 0.05). Those with the lowest socioeconomic positions had, on average, lower OOPEs; such spending increased as socioeconomic position improved.

Table 6 shows the multivariate linear regression models (that modeled the amount of OOPEs) and a logistic regression model (that modeled the likelihood of incurring OOPEs). The linear regression models demonstrated that higher age, a greater number of dental visits, a higher educational level on the part of the mother, and household car ownership were associated with an increase in OOPEs; in contrast, making curative or preventive dental visits and being affiliated with *Seguro Popular* diminished OOPEs. The logistic regression model showed that experiencing dental pain and receiving curative dental services were associated with belonging to higher socioeconomic levels and increased the likelihood of incurring OOPEs; on the other hand, SP affiliates demonstrated the lowest likelihood of incurring OOPEs. 

Table 7 shows the multivariate linear regression models that modeled the amount of OOPEs incurred by families, excluding those that reported no OOPEs (18% of households). Results were similar to those observed in the model that included families without OOPEs; this model indicated that higher age, a greater number of dental visits, a higher educational level of mothers, and household car ownership were associated with an increase in OOPEs. In contrast, making curative or preventive dental visits, being affiliated with *Seguro Popular* or the Social Security system enjoyed by the armed forces and the state oil company (*Petroleos Mexicanos* [*PEMEX*]) reduced OOPEs. 

## 4. Discussion

Our findings demonstrate that certain oral health, sociodemographic and financial features are associated with a higher likelihood of incurring OOPEs for dental care among schoolchildren attending public schools in the Mexican state of Hidalgo. Poor oral health and lacking health insurance coverage are associated with higher OOPEs. It is worth emphasizing that Mexico launched the Social Protection System in Health (SPSS by its Spanish initials) with the view of achieving universal health coverage and financially protecting people without access to Social Security services. Although the SPSS main component (the SP) has expanded the supply of health services and increased the number of interventions included in its health care package, dental services are few and limited to basic services. For example, programs offer only a limited range of curative services such as amalgam and resin fillings, tooth extractions and (occasionally) third molar surgery, as well as preventive services such as pit and fissure sealants, fluoride applications and prophylaxis. This has led to insufficient coverage for specialized treatment. Households are then forced to purchase specialized services (e.g., pedodontic, endodontic, orthodontic, prostheses and surgery care) in the private sector, leading to a significant economic impact for people with more numerous or more severe oral health problems [8]. On the other hand, we found 18.35% of people reporting no OOPEs. The two linear regression models did not differ in terms of significant variables in the final model, nor in the strength of association. Estimates did differ one from the other because households who reported no OOPEs were eliminated. It is noteworthy that, of the total households reporting zero expenditures, the majority reported that they had some form of health insurance (73.57%).

Our study indicates that the poorest households have the lowest OOPEs on dental care. Evidence exists that the use of medical and dental health services is limited when access to health services depends on the ability to pay at point of service [9,10,11,12,13,33]. Although poor people often experience disproportionate levels of oral disease [2,34,35,36,37,38,39,40,41,42], this population group is less likely to visit a dentist owing to financial barriers [43]. This state of affairs not only worsens their oral health situation; it also increases the risk of financial hardship and catastrophic expenses as a result of oral health problems [44,45]. OOPEs are one of the least efficient and most inequitable ways of financing health care [46].

Our findings also show that those with a higher demand for care and more dental visits as well as higher age incur the highest dental OOPEs. These expenses may therefore constitute a financial burden particularly for households with members at higher risk of disease [2,34,35,36,37,38,39,40,41,42]. If these are households with fewer resources, financial hardship may be heightened because of the need for more visits and possibly a greater need for specialized clinical services. According to our results, specialized clinical treatments more than double dental OOPEs.

The relationship between higher education and higher OOPEs has previously been documented [46]. This connection may stem from the fact that people with higher levels of education are more aware of their oral health needs and treatment options. Their preferences may include more specialized clinical services, resulting in higher overall expenses. This relationship has also been documented in other studies concerning the use of dental services in Mexico [9,10,11,12,13,14]. However, the simplest explanation is that the schooling variable is an indicator of income, and thus, potentially represents greater access to more expensive services, which cannot happen in households with fewer resources.

This study offers several insights concerning health insurance: households with some coverage exhibit lower OOPEs compared to those with no health insurance; households with access to Social Security benefits have lower OOPEs than those with insurance; and households affiliated with the *SP* yield the lowest OOPEs. The SP market is focused on the poorest sector of the population: 91% of affiliated households are in the first two income deciles [20], and thus, this population is less likely to be able to pay for private dental services. In this fashion, poorer households spend less. Households with Social Security coverage have high OOPEs because of the limited availability of public sector dental services. In addition, people with social security coverage are often employed in the formal economy and, thus, have a greater capacity to pay as compared to households affiliated with SP.

The importance of OOPEs as a source of financing for dental care expenditures has been documented in a study published by the Organization for Economic Co-operation and Development (OECD) [47], which found that 53% of total dental expenditures came from OOPEs. However, Bernabe et al. [48] showed that dental care expenses led to a 1.88-fold higher ratio of incurring catastrophic health expenses and 1.65-fold higher probability of facing impoverishment. In Mexico, this probability is 5.8 and 2.3 times greater, respectively. As no comprehensive estimates of total spending (public and private) on dental services have been developed in Mexico, the proportion of this type of expenditure financed by households is unclear. However, households have a high rate of financing overall healthcare—41.3% of the total health care expenditure in 2015 came from households via their OOPEs [49]. Considering the low level of coverage for dental services [8,14], it is reasonable to expect that trends would be similar to or even greater than OECD estimates. According to the 2016 National Occupation and Employment Survey in Mexico, 57% of the population in Hidalgo earns one-to-two minimum wages per day, or PPP US $650 to 867 per month on average. In this context, the dental OOPEs estimated here reach 8.1% to 10.7% of such income [50].

This is one of the first studies on dental OOPEs in a developing country that is at the same time an intermediate sized economy, that incorporates individual characteristics as well as various aspects related to oral health into its analysis The loose set of health care services used to address dental needs is not well characterized; the mosaic of services and systems makes it more challenging to fully identify how costs may be impacting services and OOPEs. This study has added to existing evidence in a largely unexplored area. Our work has some limitations, however. First, our findings are directly relevant only to households with school-aged children attending public schools in one of 32 Mexican states. Second, no information on household income was collected; therefore, it is not feasible to use our data to calculate what percentage of family income is spent on dental care; this precludes further quantification of the OOPE financial burden. Another limitation concerns the fact that data were collected by means of a questionnaire, which may have introduced memory bias. However, this form of data collection is used by various national health surveys in Mexico as well as in other countries. Validity and reliability of health issues through self-report may be affected by lack of precision; however, there is evidence that this approach is generally a reasonably accurate representation of clinical conditions [51]. Self-reporting health conditions is often used in epidemiologic surveillance for general and dental health [51,52,53,54]. Such approach in dental situations has been applied to periodontal disease [51,55,56], caries [57,58], tooth loss [59,60], or presence of teeth [61,62]. Future studies would allow measuring more accurately the financial burden of dental care in Mexican households. In general, Mexico should invest in crafting oral health policies based on evidence, in turn, supported by solid dental epidemiological and economic data.

## 5. Conclusions

Our study shows that households with higher school-aged children exhibiting the highest report of dental morbidity—as well as those without insurance—face the highest OOPEs. This poses a risk for household financial stability, in particular for the poorest households. Although some changes in the Mexican health system have expanded the supply of health services (particularly to the poorest population groups), more attention to dental services is required. Given the high prevalence of oral diseases in Mexico, it is imperative to provide health care packages that extend beyond the current low-cost, preventive dental services. These could include common specialized services to reduce OOPEs.

## Figures and Tables

**Table 1:** Description of the sociodemographic and health variables in the study sample.

Variables	Frequencies	Percentages
Age (children)		
6/7 years	221	29.0
8/9 years	237	31.0
10/12 years	305	40.0
Sex		
Boys	374	49.0
Girls	389	51.0
Number of household members		
<3	178	23.3
<5	456	59.8
≥6	129	16.9
Dental visits in the last year		
1	297	38.9
2	247	32.4
≥3	219	28.7
Dental pain in the last year		
No	238	31.2
Yes	525	68.8
Reason for the last dental visit		
Curative	501	65.6
Preventive	205	26.9
Specialty treatment	57	7.5
Report of oral disease		
No	591	77.5
Yes	172	22.5
Oral health perception		
Very poor/poor	85	11.1
Regular	309	40.5
Good/Very good	369	48.4
Frequency of tooth brushing		
Less than once a day	135	17.7
At least once a day	628	82.3
Oral health knowledge		
Low	257	33.7
Medium	288	37.7
High	218	28.6

**Table 2:** Description of the socioeconomic variables in the study sample.

Variables	Frequencies	Percentages
Educational level of mother		
Lower than elementary	58	7.6
Lower than middle	299	39.2
Lower than high school	249	32.6
High school and beyond	157	20.6
Educational level of father		
Lower than elementary	75	10.0
Lower than middle	221	29.5
Lower than high school	203	27.0
High school and beyond	251	33.5
Health insurance		
None	230	30.1
Social Security	368	48.2
SEDENA/SEMAR/PEMEX *	47	6.2
Private insurance	31	4.1
Seguro Popular	87	11.4
*SEP* (housing features)		
1st tertile	260	34.1
2nd tertile	265	34.7
3rd tertile	238	31.2
SEP (ownership of household appliances)		
1st tertile	255	33.4
2nd tertile	256	33.6
3rd tertile	252	33.0
Household car ownership		
No	279	36.8
Yes	480	63.2

* Health insurance carriers for the Army (*Secretaría de la Defensa Nacional, SEDENA*), the Navy (*Secretaría de Marina, SEMAR*) and the state oil company (*Petróleos Mexicanos, PEMEX*).

**Table 3:** Out-of-pocket expenditures on dental care in the school sample and their distribution by type of expense, International Dollars and Mexican Pesos, 2017.

Expenditure Items	PPP US$ Mean ± sd *	Mexican Pesos Mean ± sd
Transportation	2.97 ± 7.12	27.38 ± 65.72
Consultations	24.48 ± 37.68	225.92 ± 347.79
Treatments	31.89 ± 87.38	294.39 ± 806.48
Medications	6.23 ± 26.11	57.51 ± 240.99
Laboratory tests and radiographic studies	2.67 ± 12.31	24.65 ± 113.65
Others	1.63 ± 23.29	15.06 ± 214.97
Total average expenditure	70.17 ± 123.72	647.65 ± 1141.95

Kruskal-Wallis test *p* < 0.001, * 1 international dollar = 9.23 Mexican pesos.

**Table 4:** Out-of-pocket expenditures for dental care according to the sociodemographic and health characteristics of the schoolchildren, International Dollars and Mexican pesos, 2017.

Variables	PPP US$ Mean ± sd	Mexican Pesos Mean ± sd	*p* Value
Age (children)			
6/7 years	52.81 ± 75.80	487.45 ± 699.68	
8/9 years	75.95 ± 129.51	701.05 ± 1195.35	
10/12 years	78.18 ± 144.34	721.59 ± 1332.27	0.0794 *
Sex			
Boys	71.21 ± 132.03	657.24 ± 1,218.62	
Girls	69.13 ± 115.12	638.07 ± 1062.53	0.6230 †
Number of household members			
<3	50.29 ± 77.88	464.17 ± 718.85	
<5	77.29 ± 133.66	713.37 ± 1,233.69	
≥6	72.24 ± 135.74	666.82 ± 1,252.85	0.0792 †
Dental visits in the last year			
1	47.32 ± 70.76	436.79 ± 653.13	
2	60.08 ± 82.78	554.54 ± 764.04	0.0002 †
≥3	112.45 ± 190.48	1037.88 ± 1758.1	<0.001 ‡
Dental pain in the last year			
No	70.02 ± 145.23	646.28 ± 1,340.49	
Yes	70.17 ± 112.59	647.65 ± 1,039.25	<0.0001 *
Reason for the last dental visit			
Curative	55.19 ± 73.43	509.36 ± 677.77	
Preventive	70.32 ± 166.30	649.02 ± 1534.92	
Specialty treatment	201.31 ± 199.23	1858.06 ± 1838.89	0.0001 †
Report of oral disease			
No	64.09 ± 107.11	591.51 ± 988.59	
Yes	91.08 ± 167.33	840.71 ± 1544.5	0.6439 *
Oral health perception			
Very poor/poor	98.80 ± 141.97	911.91 ± 1310.36	
Regular	62.16 ± 139.00	573.71 ± 1282.98	
Good/Very good	70.17 ± 103.25	647.65 ± 952.99	0.0001 †
Frequency of tooth brushing			
Less than once a day	76.84 ± 124.46	709.27 ± 1148.79	
At least once a day	68.68 ± 123.57	633.96 ± 1140.58	0.5433 *
Oral health knowledge			
Low	52.22 ± 71.06	481.97 ± 655.87	
Medium	97.17 ± 127.43	896.85 ± 1176.18	
High	97.91 ± 158.43	903.7 ± 1462.35	0.0065 †

* Mann-Whitney, † Kruskal-Wallis, ‡ non-parametric test for trends.

**Table 5:** Out-of-pocket expenditures on dental care according to socioeconomic characteristics, International Dollars and Mexican pesos, 2017.

Variables	PPP US Mean ± sd	Mexican Pesos Mean ± sd	*p* Value
Educational level of mother			
Lower than elementary	31.60 ± 35.31	291.65 ± 325.88	
Lower than middle	81.89 ± 136.33	755.82 ± 1258.33	
Lower than high school	62.16 ± 92.42	573.71 ± 853.04	
High school and beyond	74.77 ± 155.47	690.10 ± 1434.96	0.0617 †
Educational level of father			
Lower than elementary	94.79 ± 145.82	874.94 ± 1345.96	
Lower than middle	60.08 ± 93.46	554.54 ± 862.62	
Lower than high school	57.85 ± 118.08	534.00 ± 1089.91	0.026 ‡
High school and beyond	84.71 ± 143.30	781.84 ± 1322.69	0.0080 †
Health insurance ^1^			
None	85.15 ± 149.38	785.94 ± 1378.82	
Social Security	74.32 ± 127.87	685.99 ± 1180.28	
SEDENA/SEMAR/PEMEX	48.51 ± 48.66	447.74 ± 449.11	
Private insurance	49.40 ± 40.65	455.96 ± 375.17	
Seguro Popular	32.04 ± 43.32	295.76 ± 399.82	0.0034 †
SEP (housing features)			
1st tertile	64.98 ± 109.93	599.73 ± 1014.61	
2nd tertile	61.12 ± 94.79	564.13 ± 874.94	0.028 ‡
3rd tertile	85.74 ± 159.92	791.42 ± 1476.04	0.0007 †
*SEP* (ownership of household goods)			
1st tertile	65.57 ± 104.73	605.2 ± 966.68	
2nd tertile	60.67 ± 102.95	560.02 ± 950.25	0.048 ‡
3rd tertile	84.41 ± 155.62	779.10 ± 1436.33	0.0001 †
Household car ownership			
No	44.21 ± 60.97	408.03 ± 562.76	
Yes	85.45 ± 146.72	788.68 ± 1354.18	0.0001 *

^1^ Health insurance carriers for the Army (*Secretaría de la Defensa Nacional, SEDENA*), the Navy (*Secretaría de Marina, SEMAR*), and the national oil company (*Petróleos Mexicanos, PEMEX*), * Mann-Whitney, † Kruskal-Wallis, ‡ non-parametric test for trends.

**Table 6:** Multivariate linear regression models (that modeled the amount of OOPEs) and a logistic regression model (that modeled the likelihood of incurring OOPEs).

Variables	Linear Regression Coefficient	95% CI	Logistic Regression Odds Ratio	95% CI
Age (children)				
6/7 years	1 *		1	
8/9 years	155.21 †	36.81–273.61	1.18 n/s	0.76–1.86
10/12 years	229.89 ‡	77.53–382.25	1.05 n/s	0.70–1.55
Dental visits in the last year				
1	1 *			
2	48.83 n/s	−107.60–205.28		
≥3	311.11 †	85.57–536.66	not included	
Dental pain in the last year				
No			1 *	
Yes	not included		2.25 §	1.49–3.39
Reason for the last dental visit				
Curative	−783.38 ‡	−1187.1–−235.1	2.48 ‡	1.37–4.47
Preventive	−711.15 §	−1146.9–−419.8	1 *	
Specialty treatment	1 *		2.69 n/s	0.63–11.48
Educational level of mother				
Lower than elementary	1 *			
Lower than middle	265.76 §	159.21–372.32		
Lower than high school	129.68 ‡	36.08–223.28		
High school and beyond	116.61 †	9.96–223.26	not included	
Health insurance ^1^				
None	1 *		1 *	
Social Security	−23.96 n/s	−124.69–76.75	0.67 n/s	0.43–1.05
*SEDENA/SEMAR/PEMEX*	−234.92 n/s	−479.05–9.21	1.11 n/s	0.45–2.69
Private insurance	−81.27 n/s	−211.87–49.31	1.23 n/s	0.45–3.38
*Seguro Popular*	−210.27 ‡	−315.09–−105.45	0.38§	0.26–0.53
SEP (housing features)				
1st tertile			1 *	
2nd tertile			4.91 §	2.21–10.94
3rd tertile	not included		2.47 ‡	1.23–4.95
Household car ownership				
No	1 *			
Yes	179.85 ‡	65.12–294.58	not included	

^1^ Health insurance carriers for the Army (*Secretaría de la Defensa Nacional, SEDENA*), the Navy (*Secretaría de Marina, SEMAR*), and the national oil company (*Petróleos Mexicanos, PEMEX*), † *p* < 0.05, ‡ *p* < 0.01, § *p* < 0.001, n/s = Not significant, note: models adjusted by variables in the table and sex

**Table 7:** Multivariate linear regression models that modeled the amount of OOPEs incurred by families that reported no OOPEs (*n* = 620).

Variables	Coefficient	95% CI
Age (children)		
6/7 years	1 *	
8/9 years	170.32 †	28.79–311.85
10/12 years	315.55 ‡	120.76–510.34
Dental visits in the last year		
1	1 *	
2	63.92 ^n/s^	−104.84–232.69
≥3	359.38 ‡	133.01–585.74
Reason for the last dental visit		
Curative	−914.72 §	−1123.70–−705.74
Preventive	−713.92 ‡	−1071.62–−356.23
Specialty treatment	1 *	
Educational level of mother		
Lower than elementary	1 *	
Lower than middle	313.93 ‡	166.36–461.50
Lower than high school	122.10 †	8.80–235.39
High school and beyond	310.53 ‡	153.83–467.23
Health insurance ^1^		
None	1 *	
Social Security	8.16 n/s	−143.68–160.01
SEDENA/SEMAR/PEMEX	−270.95 †	−501.05–−40.86
Private insurance	46.97 n/s	−201.46–107.52
Seguro Popular	−221.93 †	−388.46–−55.39
Household car ownership		
No	1 *	
Yes	144.64 ‡	9.25–280.03

^1^ Health insurance carriers for the Army (*Secretaría de la Defensa Nacional, SEDENA*), the Navy (*Secretaría de Marina, SEMAR*), and the national oil company (*Petróleos Mexicanos, PEMEX*), † *p* < 0.05, ‡ *p* < 0.01, § *p* < 0.001, n/s = Not significant, note: models adjusted by variables in the table and sex.

## References

[1] Guizar J.M., Muñoz N., Amador N., Garcia G. (2016). Association of Alimentary Factors and Nutritional Status with Caries in Children of Leon, Mexico. Oral. Health Prev. Dent..

[2] López-Gómez S.A., Villalobos-Rodelo J.J., Ávila-Burgos L., Casanova-Rosado J.F., Vallejos-Sánchez A.A., Lucas-Rincón S.E., Patiño-Marín N., Medina-Solís C.E. (2016). Relationship between premature loss of primary teeth with oral hygiene, consumption of soft drinks, dental care, and previous caries experience. Sci. Rep..

[3] García-Pérez A., Irigoyen-Camacho M.E., Borges-Yáñez A. (2013). Fluorosis and dental caries in Mexican schoolchildren residing in areas with different water fluoride concentrations and receiving fluoridated salt. Caries Res..

[4] Taboada-Aranza O., Rodríguez-Nieto K. (2018). Prevalence of plaque and dental decay in the first permanent molar in a school population of south Mexico City. Bol. Med. Hosp. Infant Mex..

[5] Secretaría de Salud (2006). Encuesta Nacional de Caries 2001.

[6] Guido J.A., Martinez-Mier E.A., Soto A., Eggertsson H., Sanders B.J., Jones J.E., Weddell J.A., Villanueva Cruz I., Anton de la Concha J.L. (2011). Caries prevalence and its association with brushing habits, water availability, and the intake of sugared beverages. Int. J. Paediatr. Dent..

[7] Reisine S.T. (1981). Theoretical considerations in formulating sociodental indicators. Soc. Sci. Med. A.

[8] Pérez-Núñez R., Medina-Solís C.E., Maupomé G., Vargas-Palacios A. (2006). Factors associated with dental health care coverage in Mexico: Findings from the National Performance Evaluation Survey 2002–2003. Community Dent. Oral. Epidemiol..

[9] Medina-Solís C.E., Casanova-Rosado A.J., Casanova-Rosado J.F., Vallejos-Sánchez A.A., Maupomé G., Ávila-Burgos L. (2004). Socioeconomic and dental factors associated with the use of dental services in schoolchildren in Campeche, Mexico. Bol. Med. Hosp. Infant Mex..

[10] Medina-Solís C.E., Maupomé G., Ávila-Burgos L., Casanova-Rosado J.F., Vallejos-Sánchez A.A., Segovia-Villanueva A. (2004). Use of dental health services for children under 5 years of age with social security. Rev. Mex. Pediatr..

[11] Medina-Solís C.E., Maupomé G., Ávila-Burgos L., Hijar-Medina M., Segovia-Villanueva A., Pérez-Núñez R. (2006). Factors influencing the use of dental health services by preschool children in Mexico. Pediatr. Dent..

[12] Medina-Solís C.E., Villalobos-Rodelo J.J., Márquez-Corona M.L., Vallejos-Sánchez A.A., López-Portillo-Núñez C., Casanova-Rosado A.J. (2009). Socioeconomic inequalities in the use of oral health services: Study in Mexican schoolchildren from 6 to 12 years of age. Cad. Saude Publica.

[13] Pontigo-Loyola A.P., Medina-Solís C.E., Márquez-Corona M.L., Vallejos-Sánchez A.A., Minaya-Sánchez M., Escoffié-Ramírez M. (2012). Influence of predisposing, enabling, and health care need variables on the use of dental health services among Mexican adolescents from a semi-rural location. Gac. Med. Mex..

[14] Jiménez-Gayosso S.I., Medina-Solís C.E., Lara-Carrillo E., Scougal-Vilchis R.J., de la Rosa-Santillana R., Márquez-Rodríguez S., Mendoza-Rodríguez M., de Jesús Navarrete-Hernández J. (2015). Socioeconomic inequalities in oral health service utilization any time in their lives for Mexican schoolchildren from 6 to 12 years old. Gac. Med. Mex..

[15] Comisión Nacional de Protección en Salud Catálogo Universal de Servicios de Salud Causes 2016. http://www.documentos.seguro-popular.gob.mx/dgss/CAUSES2016.pdf.

[16] Gutiérrez J.P., Rivera-Dommarco J., Shamah-Levy T., Villalpando-Hernández S., Franco A., Cuevas-Nasu L., Romero-Martínez M., Hernández-Ávila M. (2012). Encuesta Nacional de Salud y Nutrición 2012. Resultados Nacionales. https://ensanut.insp.mx/informes/ENSANUT2012ResultadosNacionales.pdf.

[17] Frenk J., Gómez Dantés O. (2015). El sistema de Salud Mexicano.

[18] Ávila-Burgos L., Serván-Mori E., Wirtz V.J., Sosa-Rubí S.G., Salinas-Rodríguez A. (2013). Efectos del Seguro Popular sobre el gasto en salud en hogares mexicanos a diez años de su implementación. Salud Publica Mex..

[19] Chemor-Ruiz A., Ochmann-Ratsch A.E., Alamilla-Martínez G.A. (2018). Mexico’s Seguro Popular: Achievements and Challenges. Health Syst. Reform..

[20] Government of the Republic (2018). Social Protection in Health System: Results Report January–December 2017.

[21] Sosa-Rubí S.G., Galárraga O., Harris J.E. (2009). Heterogeneous impact of the “Seguro Popular” program on the utilization of obstetrical services in Mexico, 2001-2006: A multinomial probit model with a discrete endogenous variable. J. Health Econ..

[22] Escoffié-Ramirez M., Ávila-Burgos L., Baena-Santillan E.S., Aguilar-Ayala F., Lara-Carrillo E., Minaya-Sánchez M., Mendoza-Rodríguez M., de Lourdes Márquez-Corona M., Medina-Solís C.E. (2017). Factors associated with dental pain in Mexican schoolchildren aged 6 to 12 years. Biomed. Res. Int..

[23] Medina-Solís C.E., Pontigo-Loyola A.P., Maupomé G., Lamadrid-Figueroa H., Loyola-Rodriguez J.P., Hernández-Romano J., Villalobos-Rodelo J.J., Márquez-Corona M.L. (2008). Dental fluorosis prevalence and diagnostic test using Dean’s index based on 6 teeth, and on 28 teeth. Clin. Oral. Investig..

[24] INSP El Consumo de Azúcar en México y la Nueva Directriz de la OMS para su Reducción Global. https://www.insp.mx/epppo/blog/3609-consumo-azucar-mexico-nueva-directriz-oms.html.

[25] WHO (1997). Chapter 1: Organization of a Basic Oral Health Survey. Oral Health Survey—Basic Methods.

[26] National Institute of Statistic and Geography (INEGI) Inflation Calculator. http://www.inegi.org.mx/sistemas/indiceprecios/CalculadoraInflacion.aspx.

[27] OECD Data. Purchasing Power Parities. http://www.imf.org/external/np/fin/data/param_rms_mth.aspx.

[28] World Bank Data. Purchasing Power Parities. https://datos.bancomundial.org/indicador/PA.NUS.PPP?view=map.

[29] Sun G.W., Shook T.L., Kay G.L. (1996). Inappropriate use of bivariable analysis to screen risk factors for use in multivariable analysis. J. Clin. Epidemiol..

[30] Bagley S.C., White H., Golomb B.A. (2001). Logistic regression in the medical literature: Standards for use and reporting, with particular attention to one medical domain. J. Clin. Epidemiol..

[31] Williams R.L. (2000). A note on robust variance estimation for cluster-correlated data. Biometrics.

[32] Fernández-Barrera M.Á., Medina-Solís C.E., Casanova-Rosado J.F., Mendoza-Rodríguez M., Escoffié-Ramírez M., Casanova-Rosado A.J., de Jesús Navarrete-Hernández J., Maupomé G. (2016). Contribution of prosthetic treatment considerations for dental extractions of permanent teeth. PeerJ.

[33] Alkenbrack S., Lindelow M. (2015). The impact of community-based health insurance on utilization and out-of-pocket expenditures in Lao People’s Democratic Republic. Health Econ..

[34] Villalobos-Rodelo J.J., Medina-Solís C.E., Maupomé G., Vallejos-Sánchez A.A., Lau-Rojo L., de León-Viedas M.V. (2007). Socioeconomic and sociodemographic variables associated with oral hygiene status in Mexican schoolchildren aged 6 to 12 years. J. Periodontol..

[35] Islas-Granillo H., Borges-Yañez S.A., Medina-Solís C.E., Galan-Vidal C.A., Navarrete-Hernández J.J., Escoffié-Ramirez M., Maupome G. (2014). Salivary Parameters (Salivary Flow, pH and Buffering Capacity) in Stimulated Saliva of Mexican Elders 60 Years Old and Older. West Indian Med. J..

[36] Medina-Solís C.E., Maupomé G., Pelcastre-Villafuerte B., Avila-Burgos L., Vallejos-Sánchez A.A., Casanova-Rosado A.J. (2006). Socioeconomic inequalities in oral health: Dental caries in 6 to 12 year-old children. Rev. Investig. Clin..

[37] Herrera M.S., Medina-Solís C.E., Robles-Bermeo N.L., Minaya-Sánchez M., Alonso-Sánchez C.C., Lara-Carrillo E., Mendoza-Rodríguez M., Bayardo-González R.A. (2017). Consultation for dental extraction in Nicaraguan children: An approach to oral care needs. Pediatr.

[38] van der Tas J.T., Kragt L., Elfrink M.E.C., Bertens L.C.M., Jaddoe V.W.V., Moll H.A., Ongkosuwito E.M., Wolvius E.B. (2017). Social inequalities and dental caries in six-year-old children from the Netherlands. J. Dent..

[39] Shin H.S. (2018). Social gradients in oral health status in Korea population. Arch. Oral. Biol..

[40] Andrade F.B., Antunes J.L.F., Souza Junior P.R.B., Lima-Costa M.F., Oliveira C. (2018). Life course socioeconomic inequalities and oral health status in later life: ELSI-Brazil. Rev. Saude Publica.

[41] Costa S.M., Martins C.C., Pinto M.Q.C., Vasconcelos M., Abreu M.H.N.G. (2018). Socioeconomic factors and caries in people between 19 and 60 years of age: An update of a systematic review and meta-analysis of observational studies. Int. J. Environ. Res. Public Health.

[42] Kailembo A., Preet R., Stewart-Williams J. (2018). Socioeconomic inequality in self-reported unmet need for oral health services in adults aged 50 years and over in China, Ghana, and India. Int. J. Equity Health.

[43] Kailembo A., Quiñonez C., Lopez-Mitnik G.V., Weintraub J.A., Stewart-Williams J., Preet R., Iafolla T., Dye B.A. (2018). Income and wealth as correlates. Of socioeconomic disparity in dentist visits among adults aged 20 years and over in the United States, 2011–2014. BMC Oral. Health..

[44] Kim Y., Yang B. (2011). Relationship between catastrophic health expenditures and household incomes and expenditure patterns in South Korea. Health Policy.

[45] Sun X., Bernabe E., Liu X., Gallagher J.E., Zheng S. (2016). Determinants of Catastrophic Dental Health Expenditure in China. PLoS ONE.

[46] Knaul F.M., Frenk J. (2005). Health insurance in Mexico: Achieving universal coverage through structural reform. Health Aff..

[47] OECD (2013). Health at Glance.

[48] Bernabé E., Masood M., Vujicic M. (2017). The impact of out-of-pocket payments for dental care on household finances in low and middle income countries. BMC Public Health.

[49] Health Ministry DGIS. Financial Resources. Expenditure on Health in the National Health System. http://www.dgis.salud.gob.mx/contenidos/sinais/gastoensalud_gobmx.html.

[50] González E. (2016). Salaries in Hidalgo. Milenio. http://www.milenio.com/opinion/eduardo-gonzalez/intelecto-opuesto/salarios-en-hidalgo.

[51] Quiroz V., Reinero D., Hernández P., Contreras J., Vernal R., Carvajal P. (2017). Development of a self-report questionnaire designed for population-based surveillance of gingivitis in adolescents: Assessment of content validity and reliability. J. Appl. Oral. Sci..

[52] Jamieson L.M., Thomson W.M., McGee R. (2004). An assessment of the validity and reliability of dental self-report items used in a National Child Nutrition Survey. Community Dent. Oral. Epidemiol..

[53] Schluter P.J., Durward C., Cartwright S., Paterson J. (2007). Maternal self-report of oral health in 4-year-old Pacific children from South Auckland, New Zealand: Findings from the Pacific Islands Families Study. J. Public Health Dent..

[54] Blizniuk A., Ueno M., Zaitsu T., Kawaguchi Y. (2017). Association between self-reported and clinical oral health status in Belarusian adults. J. Investig. Clin. Dent..

[55] García-Pérez Á., Borges-Yáñez S.A., Jiménez-Corona A., Jiménez-Corona M.E., Ponce-de-León S. (2016). Self-report of gingival problems and periodontitis in indigenous and non-indigenous populations in Chiapas, Mexico. Int. Dent. J..

[56] Abbood H.M., Hinz J., Cherukara G., Macfarlane T.V. (2016). Validity of self-reported periodontal disease: A systematic review and meta-analysis. J. Periodontol..

[57] Levin L., Shpigel I., Peretz B. (2013). The use of a self-report questionnaire for dental health status assessment: A preliminary study. Br. Dent. J..

[58] Silva A.E., Menezes A.M., Assunção M.C., Gonçalves H., Demarco F., Vargas-Ferreira F., Peres M. (2014). Validation of self-reported information on dental caries in a birth cohort at 18 years of age. PLoS ONE.

[59] Haugejorden O., Klock K.S., Trovik T.A. (2003). Incidence and predictors of self-reported tooth loss in a representative sample of Norwegian adults. Community Dent. Oral. Epidemiol..

[60] Gilbert G.H., Chavers L.S., Shelton B.J. (2002). Comparison of two methods of estimating 48-month tooth loss incidence. J. Public Health Dent..

[61] Matsui D., Yamamoto T., Nishigaki M., Miyatani F., Watanabe I., Koyama T., Ozaki E., Kuriyama N., Kanamura N., Watanabe Y. (2016). Validity of self-reported number of teeth and oral health variables. BMC Oral. Health.

[62] Ueno M., Shimazu T., Sawada N., Tsugane S., Kawaguchi Y. (2018). Validity of self-reported tooth counts and masticatory status study of a Japanese adult population. J. Oral. Rehabil..

